# Exploring the effects of cannabidiol encapsulation in liposomes on their physicochemical properties and biocompatibility

**DOI:** 10.1080/10717544.2025.2460666

**Published:** 2025-02-06

**Authors:** Inga Jurgelane, Karina Egle, Andra Grava, Dana Galkina, Margarita Brante, Maksims Melnichuks, Marite Skrinda-Melne, Girts Salms, Arita Dubnika

**Affiliations:** a Faculty of Natural Sciences and Technology, Institute of Biomaterials and Bioengineering, Riga Technical University, Riga, Latvia; b Baltic Biomaterials Centre of Excellence, Riga Technical University, Riga, Latvia; c Institute of Stomatology, Riga Stradins University, Riga, Latvia

**Keywords:** Liposomes, cannabidiol, drug delivery, nanosized drugs, increased bioavailability

## Abstract

Cannabidiol (CBD) is recognized for its therapeutic properties in various conditions. However, CBD’s limited water solubility and sensitivity to environmental stresses hinder its efficacy and bioavailability. Encapsulation in drug delivery systems, particularly liposomes, offers a promising solution. This study aims to prepare CBD-containing liposomes using commercially used lipids distearoyl phosphatidylcholine (DSPC) and dipalmitoyl phosphatidylcholine (DPPC), and 1,2 distearoyl-sn-glycero-3 phosphoethanolamine-N-[carbonyl-amino(polyethylene glycol)-4300] (ammonium salt) (DSPE-PEG) and to perform *in vitro* studies – cell viability and CBD release. Liposomes were synthesized using thin-film hydration method, and characterized by Fourier-transform infrared (FT-IR) spectroscopy, dynamic light scattering (DLS), and scanning transmission electron microscopy (STEM). DLS analysis revealed that CBD incorporation reduced liposome size by 23–53%, depending on the liposomes. Encapsulation efficiency followed the order: DPPC CBD (63%) < DSPC CBD (74%) < DSPC DPPC CBD (81%) < DSPC DSPE-PEG CBD (87%). CBD release profiles indicated that DPPC CBD liposomes released the highest CBD amount initially, while DSPC DSPE-PEG CBD exhibited sustained release, achieving 79% release over 504 h. *In vitro* cell viability tests showed that blank liposomes were non-cytotoxic. However, CBD-loaded liposomes significantly reduced cell viability for defined type of CBD containing liposomes. The inclusion of DSPE-PEG improved encapsulation efficiency and liposome stability, making DSPC DSPE-PEG CBD liposomes more suitable for long-term CBD release. Compared to other studies, encapsulation of CBD in liposomes enhances its bioavailability, allowing lower concentrations of CBD to be directly delivered to cells, resulting in observable changes in cell viability.

## Introduction

Cannabidiol (CBD) is recognized for its therapeutic properties in various indications (Wade et al., 2003; Mannila et al., 2007; Villanueva et al., 2022; Wang et al., 2022) and can be administered via multiple routes including enteral, parenteral, intranasal, inhalation, and transdermal methods (Stella et al., 2021). Due to its antibacterial, anti-inflammatory and antioxidant properties (Sunda & Arowolo, 2020), CBD is being widely investigated for the treatment of oral diseases such as oral cancer, gingivitis, and periodontitis (Hu et al., 2024). CBD can speed up wound healing (Klein et al., 2018) and relieve pain caused by chemotherapy (Heider et al., 2022). Recent research has demonstrated that CBD suppresses the viability of human tongue squamous carcinoma HSC-3 cells by inducing DNA damage, thereby inhibiting the growth of oral cancer (Billi et al., 2025).

Nevertheless, the primary challenge in using CBD is its limited water solubility (0.01 mg/mL), hindering direct oral consumption and dispersion in hydrophilic matrices. Consequently, only 6% of the orally consumed dose escapes hepatic first-pass metabolism, resulting in oral bioavailability values of less than 19% (Lastres-Becker et al., 2005; Zapata et al., 2023). Additionally, CBD shows intrinsic sensitivity to environmental stresses and can degrade when exposed to heat, light, and oxygen. Therefore, enhancing the efficacy and stability of CBD is crucial.

Encapsulation of hydrophobic substances in drug delivery systems offers a promising solution to improve drug stability and ensure more controllable and predictable release (Millar et al., 2020). Current drug delivery research focuses on nanosized systems for their broad treatment and diagnostic applications (Zapata et al., 2023), with lipids being particularly effective due to their easy modification through processes like acidolysis, alcoholysis, hydrolysis, and esterification (Baeza-Jiménez et al., 2014). Liposomes are spherical drug delivery vehicles composed of phospholipids and often cholesterol (CH). They offer significant advantages over other delivery methods, such as mimicking natural cell membranes to transfer active compounds directly in cell (He et al., 2019). There are various theories on liposome interaction with the cell membrane, namely, adsorption, endocytosis, fusion, and lipid exchange (Gandek et al., 2023).

Both hydrophilic and hydrophobic drugs can be incorporated in liposomes, with hydrophilic drugs housed inside the core and hydrophobic drugs, like CBD, layered within the liposome double layer. The main ways liposomes can be administered are ocular, oral, pulmonary, and transdermal (Çağdaş et al., 2014). In recent years, various studies are aimed at detailed research of advancing CBD drug delivery systems (Franzè et al., 2022; Fu et al., 2022; Kok et al., 2022; Shilo-Benjamini et al., 2022; Tabboon et al., 2022; Zapata et al., 2023). Existing production of suitable CBD carriers is expensive, time, and energy-consuming. Fraguas-Sánchez et al. (2020) conducted a synthesis of CBD microcapsules consisting of multiple time-consuming stages using a separation membrane and various reagents that can increase production costs.

Until now, there are limited studies on CBD-containing liposomes. Zapata et al. prepared CBD and soy lecithin nanoliposomes intended for oral application and performed drug release studies in buccal, stomach, and duodenal conditions (Zapata et al., 2023). CBD release kinetics was investigated for liposomes prepared from soybean phosphatidylcholine and 20(S)-protopanaxadiol and these liposomes were used also for *in vivo* studies on murine breast tumors (Fu et al., 2022). Shilo-Benjamini et al. (2022) used liposomal formulation of hydrogenated soy phosphatidylcholine and CBD for *in vivo* studies as analgesic treatment for dogs with chronic pain. CBD plasma profile was measured during 28 days after the injection of liposomal CBD (Shilo-Benjamini et al., 2022). Additionally, liposomal-CBD formulations have demonstrated minimal side effects while reducing pain and improving well-being in dogs with osteoarthritis (Shilo-Benjamini et al., 2023).

Distearoyl phosphatidylcholine (DSPC) and dipalmitoyl phosphatidylcholine (DPPC) based liposomes have been researched for encapsulation of various hydrophobic substances like ibuprofen, propofol, midazolam (Khadke et al., 2018), l-cysteine (Perrotta et al., 2016), doxorubicin (Parr et al., 1997), and glibenclamide (Maritim et al., 2021). Moreover, these lipids are used in commercial liposomal products like Arikaye (DPPC) and DaunoXome (DSPC) (Liu et al., 2022). These phospholipids also are fully saturated lipids, and research shows that liposomes composed of saturated lipids are more stable than those liposomes composed of unsaturated phospholipids (Lian & Ho, 2001). Therefore in this research, DSPC and DPPC lipids were used in liposome preparation.

The aim of this research is to prepare CBD-containing liposomes with different compositions and to evaluate the effect of CBD concentration and liposome composition on *in vitro* studies – CBD release and cell viability for future application in treatment of oral diseases.

## Materials and methods

### Materials

Pharma grade, synthetic CBD was purchased from CBDepot.eu (Teplice, Czech Republic). DSPC, DPPC, and 1,2-distearoyl-sn-glycero-3-phosphoethanolamine-N-[carbonyl-amino(polyethy­lene glycol)-4300] (ammonium salt) (DSPE-PEG) were purchased from Avanti Polar Lipids (Alabaster, AL). CH, absolute ethanol, formic acid, acetonitrile, phosphate-buffered saline (PBS) tablets, dimethyl sulfoxide (DMSO), fetal bovine serum (FBS), collagenase, trypsin 0.25% EDTA solution, penicillin–streptomycin (P/S), Cell Counting Kit-8 (CCK8), and MilliporeSigma™ Chemicon™ Mesenchymal Adipogenesis Kit were obtained from Sigma Aldrich (St. Louis, MO). StemPro^®^ Osteogenesis Differentiation Kit, StemPro^®^ Chondrogenesis Differentiation Kit and Dulbecco’s modified Eagle’s medium with l-glutamine, 4.5 g/L glucose, sodium pyruvate, and sodium bicarbonate (DMEM) was obtained from Gibco (Carlsbad, CA).

### Cell isolation

Gingiva-derived mesenchymal stem cells (GMSCs) were isolated from human patients and used for *in vitro* viability tests. This study was conducted in accordance with the Declaration of Helsinki, and the protocol was approved by the Riga Stradins University Research Ethics Committee Decision No. 6-1/12/47 (26.11.2020). Tissue samples from the patients were obtained during the placement of dental implants. Patient selection was conducted by the surgeon, with the primary criterion being that donors had no underlying health conditions. Patients provided informed consent for the use of their samples in research studies. These GMSC’s were solely obtained for the INJECT-BIO project purposes, including but not limited to this study.

Following the biopsy, the tissue sample was immediately placed into a 15 mL centrifuge tube with P/S supplemented DMEM and stored at +4 °C. The cell isolation from tissue was performed as soon as possible – no longer than 24 h after the biopsy has been collected. The biopsy sample was transferred into a Petri dish with 2 mL of collagenase diluted in DMEM (5 mg/mL) and minced into smaller fragments with sterile tweezers and scissors. The entire Petri dish content was transferred to 15 mL centrifuge tube and another 3 mL of collagenase diluted in DMEM (5 mg/mL) was added. The tube was placed into a rotator where the sample was continuously stirred with circular movements and tissue was enzymatically digested for 2 h at +37 °C. Then, the collagenase solution, containing the isolated cells, was removed from tissue fragments and transferred to a new 15 mL centrifuge tube. The tube was centrifuged (1400 rpm 5 min) to pellet cells and remove all enzyme solution from cells. The supernatant was removed and the cells were resuspended with 2 mL of cell culture media (10% of FBS, 1% of P/S, and 89% of DMEM). The cells were placed in a six-well plate into an incubator at +37 °C and 5% CO_2_ to further cultivate the obtained cells. All obtained GMSC’s were tested for osteogenic, adipogenic, and chondrogenic differentiation using StemPro^®^ Osteogenesis Differentiation Kit, MilliporeSigma™ Chemicon™ Mesenchymal Adipogenesis and StemPro^®^ Chondrogenesis Differentiation Kit, respectively.

### Liposome synthesis

**Figure 1.**
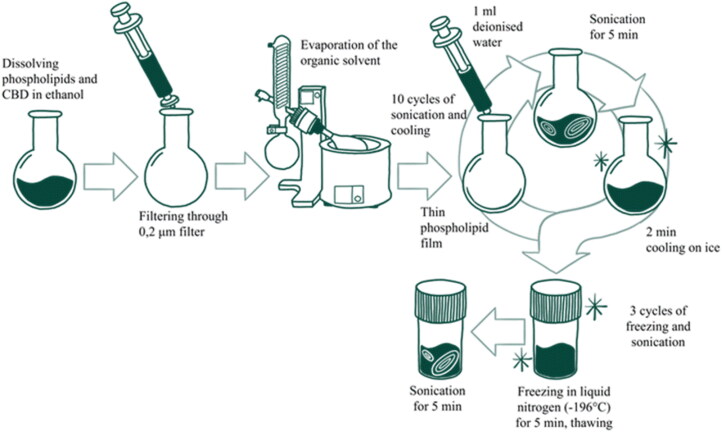
Scheme of liposome synthesis.

The liposome samples were obtained using a thin-film hydration method shown in Figure 1. The phospholipids in various compositions with or without CBD and CH were dissolved in absolute ethanol. Lipid solution was filtered through a filter with 0.2 μm pore size, to filter off any potential particles that could act as crystallization centers. Then, the solution was put in a rotary evaporator and ethanol was evaporated for about 70 min at 30 °C under 900 mBar pressure. To reduce the size, prevent aggregation and ensure higher encapsulation efficiency of obtained liposomes, the sonication method was applied (Umbarkar et al., 2021; Andra et al., 2022). Therefore, the following step was a repetitive cycle of ultrasonication with deionized water – addition of 1 mL deionized water in each cycle, 5 min of ultrasonication and 2 min of cooling on ice. After five cycles (overall 5 mL of deionized water was added), the liposomes were ultrasonicated without water addition for five cycles − 2 min of ultrasonication and 1 min of cooling on ice. All ultrasonications were performed in a 42 kHz bath ultrasonicator with a sonication intensity of 22.01 kW/m^2^ at room temperature. These parameters were chosen according to the properties of CBD, such as, relatively low degradation temperature of CBD. The ultrasonication was performed in an amber glass round bottom flask at room temperature to ensure minimal exposure to environmental conditions – light, oxygen, and increased temperature. Also, it was performed in short, intensive irradiation cycles with cooling on ice to avoid overheating and degradation of samples (Woodbury et al., 2006; Taladrid et al., 2017; Babakhanian et al., 2018; Kosović et al., 2021). This approach maintains the integrity of CBD while effectively decreasing liposome size. The last step of liposome preparation was freeze-thawing, where liposomes were put in 8 mL containers, frozen in liquid nitrogen, thawed and ultrasonicated for 5 min. This action was repeated three times. The liposomes were put in −26 °C for further analysis. The composition of prepared liposomes is shown in Table 1.

**Table 1.** Liposome composition.

Liposome sample name	Composition and molar ratio	CBD, mg
DSPC	DSPC:CH = 2:1	0
DPPC	DPPC:CH = 2:1	0
DSPC DPPC	DSPC:DPPC:CH = 1:1:1	0
DSPC DSPE-PEG	DSPC:CH:DSPE-PEG = 2:1:0.5	0
DSPC CBD	DSPC:CH = 2:1	5
DPPC CBD	DPPC:CH = 2:1	5
DSPC DPPC CBD	DSPC:DPPC:CH = 1:1:1	5
DSPC DSPE-PEG CBD	DSPC:CH:DSPE-PEG = 2:1:0.5	5

### Characterization methods

Liposomes, lipids, and CBD were characterized with Fourier-transform infrared (FT-IR) spectroscopy using Nicolet IS50 FT-IR equipped with an attenuated total reflection (ATR) sample holder iS50 ATR (Thermo Fisher Scientific, Waltham, MA). Liposomes were lyophilized using freeze dryer BETA 2-8 LSCplus (Martin Christ, Osterode, Germany). The spectra were recorded in the range of 4000–400 cm^−1^ with an accuracy of 4 cm^−1^, performing 50 sample scans.

The liposome size was determined with dynamic light scattering (DLS) analysis using Anton Paar Litesizer 500 (Anton Paar, Graz, Austria). The frozen liposome suspensions were thawed at room temperature and sonicated for 3 min. The obtained suspension was diluted 1:1000 in aqueous medium. Liposomes are assumed to have a refractive index of 1.45, an absorbance of 0.001 at temperature of 25 °C (Gardiner et al., 2014).

The morphology of the liposomes were analyzed with scanning transmission electron microscopy (STEM) using Verios 5 UC Thermo Scientific microscope (Thermo Fisher Scientific, Waltham, MA), operating at an acceleration voltage of 30 kV. Seven microliters of liposome suspension (conc. 0.5 mg/mL) was placed on a 400 mesh copper grid with formvar carbon film, allowed to adsorb for one minute and extra sample was carefully removed using a filter paper. Then, immediately, 7 μL of aqueous 2% (w/v) ammonium molybdate solution was added on top of the sample and left for 2.5 minutes. The extra solution was removed with filter paper and the samples were left to dry at room conditions from two hours to overnight.

### CBD release kinetics

The liposome samples were prepared by mixing each liposome synthesis with distilled water in ratio 1:2. Two hundred and fifty microliters of the prepared liposome samples were put inside the inserts in 24-well plates with 750 μL of PBS beneath the inserts. All plates were wrapped in foil and placed in an incubator-shaker at 37 °C, 50 rpm. The PBS was changed twice a day. The entire liposome suspension was removed from the inserts after 1, 5, 24, 48, 96, 168, 240, 336, and 504 h (21 days in total), put in a freezer at −26 °C and lyophilized.

The CBD release was determined with ultra-performance liquid chromatography (UPLC) using Acquity UPLC H-class (Waters, Milford, MA) chromatograph with UV/VIS detector Acquity TUV (Waters, Milford, MA) set at 228 nm, and Acquity UPLC BEH C18 column (1.7 µm, 2.1 × 150 mm) (Waters, Milford, MA) with a pre-column Acquity UPLC BEH C18 (1.7 µm, 2.1 × 5 mm) (Waters, Milford, MA). The chromatographic method was adapted based on another study (Therapeutic Goods Administration, 2024). The lyophilized liposome samples were dissolved in 1 mL of a diluent consisting of eluents A (0.1% formic acid in water) and B (0.1% ­formic acid in acetonitrile) in 30:70 (v/v) ratio. The samples were sonicated, centrifuged at 3000 rpm and filtered through a 0.2 µm nylon filter. A mobile phase of eluents A and B in 25:75 (v/v) ratio was used at a flow rate of 0.2 mL/min. Each sample was analyzed for eight minutes, with the column at 30 °C ± 5 °C, and sample at 10 °C ± 5 °C. The limit of quantification and limit of detection for the developed method were found to be 1.514 ± 0.125 µg/mL and 0.500 ± 0.041 µg/mL. For each series of samples, three repetitions are prepared – one from each parallel synthesis. From the obtained results, encapsulation efficiency was calculated.

### **In vitro** cell viability

The GMSC’s were seeded in 96-well plate (10^6^ cells per well) in 100 μL of cell culture medium and left overnight in incubator (at 37 °C and 5% of CO_2_) to attach to the well surface. After 24 h, the old cell culture medium was aspirated and a new cell medium with 100 μL various liposome concentrations (1, 3, 7, and 12 µg/mL) was added to the cells. For the negative control, 5% DMSO was used. The cell plates were incubated for 24, 48, 72, and 96 h. Then, the medium was aspirated and the cells were washed twice with PBS in order to remove the liposomes. Afterwards, 100 μL of 10% (v/v) CCK8 assay solution was added to cells and incubated for 1 h. The optical density of each well was measured at 450 nm with microplate reader Infinite M Nano (Tescan, Brno, Czech Republic).

### Statistical analysis

Data for CBD release were generated in triplicates and for *in vitro* cell viability six parallel samples were used. Two-tailed *t*-test analysis was used to determine the statistical significance of liposome particle size, cell viability, and CBD release, using OriginPro 9.1 software (OriginLab, Northampton, MA). Data are represented as the mean ± standard deviation.

## Results

### Characterization

**Figure 2.**
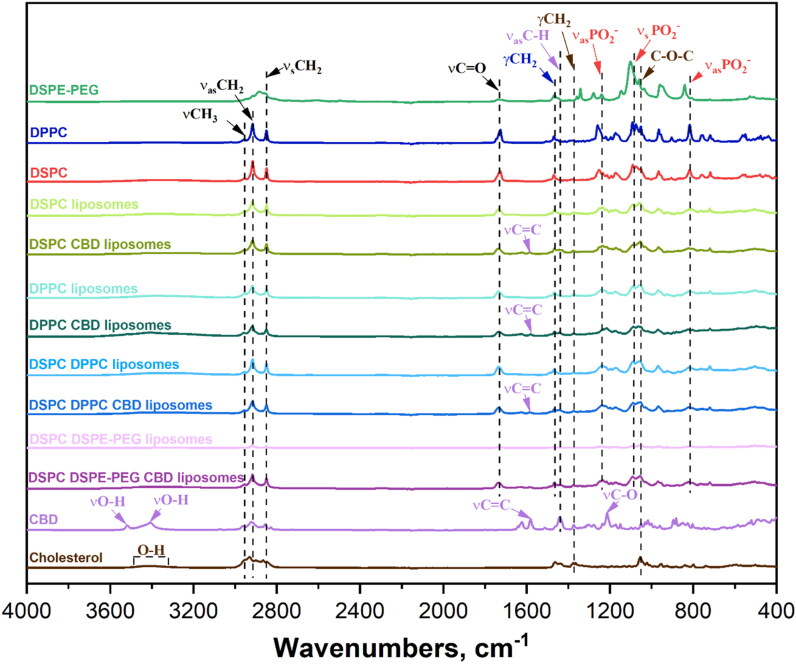
FTIR spectra of used substances and prepared liposomes in the full spectral range.

The FTIR spectra (Figure 2) show the characteristic peaks of the substances and prepared liposomes. CH characteristic peaks are at 3430 cm^−1^ (O–H bond), 2920 and 2848 cm^−1^ (C–H oscillations), 1464 cm^−1^ (C═C), 1375 cm^−1^ (O–H and C–O–H bending/stretching), and 1055 cm^−1^ (C–O vibrations). DSPC lipid peaks are at 2917–2921 cm^−1^ (CH_2_ stretching), 2849–2852 cm^−1^ (CH_2_ stretching), 1740 cm^−1^ (C═O stretching), 1085 and 1260 cm^−1^ (PO_2_ stretching), and 820 cm^−1^ (O–P–O stretching). DPPC lipid peaks are similar to DSPC but differ in intensity, because they do not differ from each other with functional groups, only with the length of the carbon chain. DSPE-PEG lipid peaks are at 2900 cm^−1^ (C–H stretching), 1730 cm^−1^ (C═O stretching), and 1600 cm^−1^ (N–H bending). CBD peaks are at 3513 and 3401 cm^−1^ (O–H stretching), 3000 cm^−1^ (C–H vibrations), 2914 cm^−1^ (methyl and methylene groups), 1575 cm^−1^ (C═C stretching), and 1216 cm^−1^ (C–O stretching). The liposome spectra reflect their constituent substances, with DSPC being the dominant. CBD liposomes show both lipid and CH bonds, with less intense CBD peaks due to its lower concentration.

**Figure 3.**
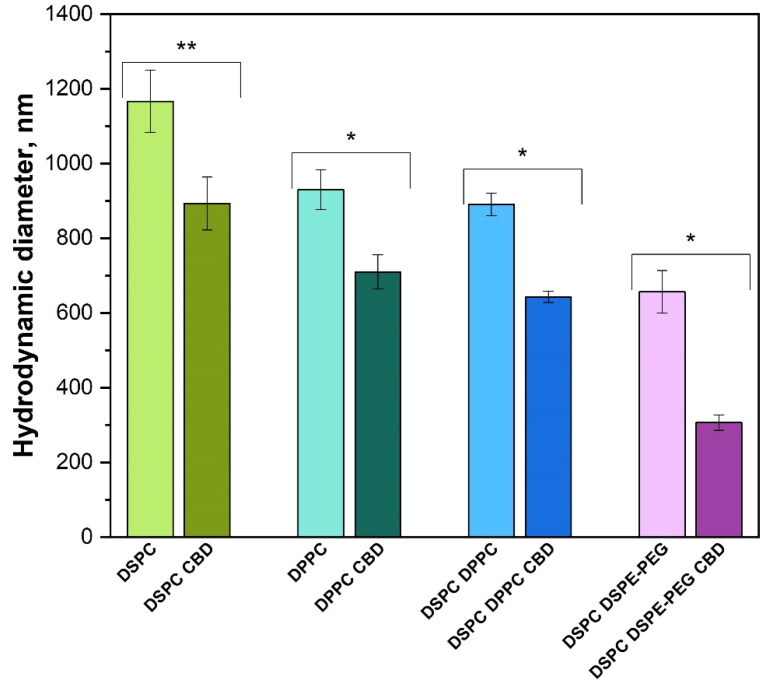
Hydrodynamic diameter of liposomes (*p < .01, **p < .05; *n* = 3).

The hydrodynamic diameter of the liposome samples are shown in Figure 3. It should be noted that DLS analysis for inhomogeneous suspensions provides only a rough estimate of particle sizes. The liposome size increased in the direction DSPC DSPE-PEG < DSPC DPPC < DPPC < DSPC from 657 ± 57 to 1167 ± 83 nm, but there are no significant differences between the sizes of DPPC and DSPC DPPC liposomes. The incorporation of CBD into liposomes noticeably decreased the size for all liposome formulations by 23–53%, maintaining the same size trend as for liposomes without CBD.

**Figure 4.**
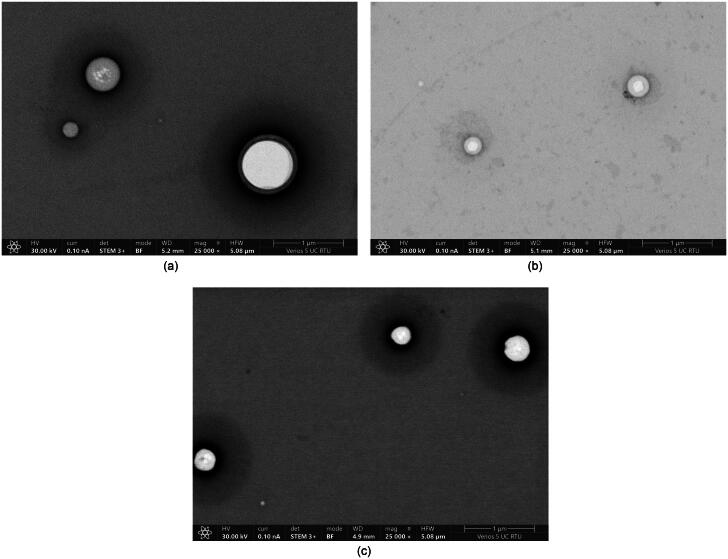
STEM images: (A) DSPC liposomes dried overnight, (B) DSPC liposomes with incorporated CBD dried overnight, and (C) DSPC liposomes dried for 2 h.

In STEM images, it can be seen that the liposomes are uniform with smooth and homogeneous surfaces and spherical shapes (Figure 4(A)). The minimal halo effect surrounding the liposomes indicates the purity and uniformity of the liposome population. The addition of CBD causes noticeable differences (Figure 4(B)) – smaller size and more pronounced halo effect than for liposomes without CBD. It indicates that the presence of CBD changes the surface properties or the presence of additional material associated with the liposome surfaces. This suggests that the incorporation of CBD also influences the structural integrity and stability of liposomes. The lighter region in the center of the liposomes is due to the overnight drying process. Liposomes dried for only two hours did not show this characteristic (Figure 4(C)), suggesting that the lipid bilayer burst during extended drying.

### CBD encapsulation efficiency and release

The encapsulation efficiency of CBD increased in direction DPPC CBD (63%) < DSPC CBD (74%) < DSPC DPPC CBD (81%) < DSPC DSPE-PEG CBD (87%). Similar results were observed in other research (Anderson & Omri, 2004; Maritim et al., 2021), where lipids with longer acyl chains achieved higher encapsulation efficiency. The highest efficiency was achieved for DSPC DSPE-PEG CBD liposomes, probably due to the improved stability by the addition of PEGylated lipid (Izumi et al., 2024).

**Figure 5.**
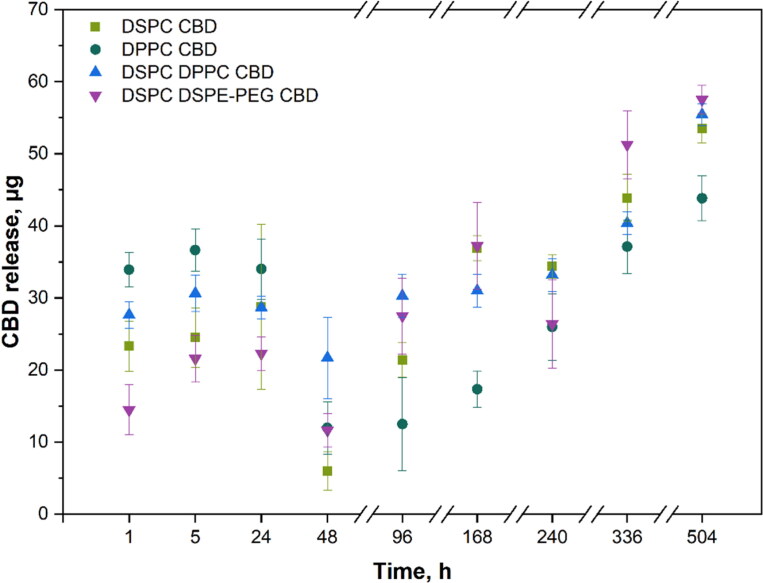
CBD release kinetics from DSPC CBD, DPPC CBD, DSPC DPPC CBD, and DSPC DSPE-PEG CBD liposomes in PBS for 504 h; *n* = 3.

The release profiles of CBD from different liposomes were evaluated over a 504 h period and are shown in Figure 5. DPPC CBD liposomes released the highest CBD amount in the 1 h − 34 ± 2 µg, which is 63% of the encapsulated CBD amount (CBD at 0 h), but after 48 h a slower release over time was observed, achieving 83% (44 ± 3 µg) of the encapsulated CBD at 504 h. On the other hand, DSPC DSPE-PEG CBD released the lowest CBD amount in the 1 h − 15 ± 4 µg, which is only 20% of the encapsulated CBD amount, but after 96 h a higher cumulative release over time was observed, yet achieving only 79% (58 ± 2 µg) at 504 h.

The release profiles of CBD from different liposomes were evaluated over a 504 h period and are shown in Figure 5. DPPC CBD liposomes released the highest CBD amount in the 1 h − 34 ± 2 µg, which is 63% of the encapsulated CBD amount (CBD at 0 h), but after 48 h a slower release over time was observed, achieving 83% (44 ± 3 µg) of the encapsulated CBD at 504 h. On the other hand, DSPC DSPE-PEG CBD released the lowest CBD amount in the 1 h − 15 ± 4 µg, which is only 20% of the encapsulated CBD amount, but after 96 h a higher cumulative release over time was observed, yet achieving only 79% (58 ± 2 µg) at 504 h.

### **In vitro** cell viability

**Figure 6.**
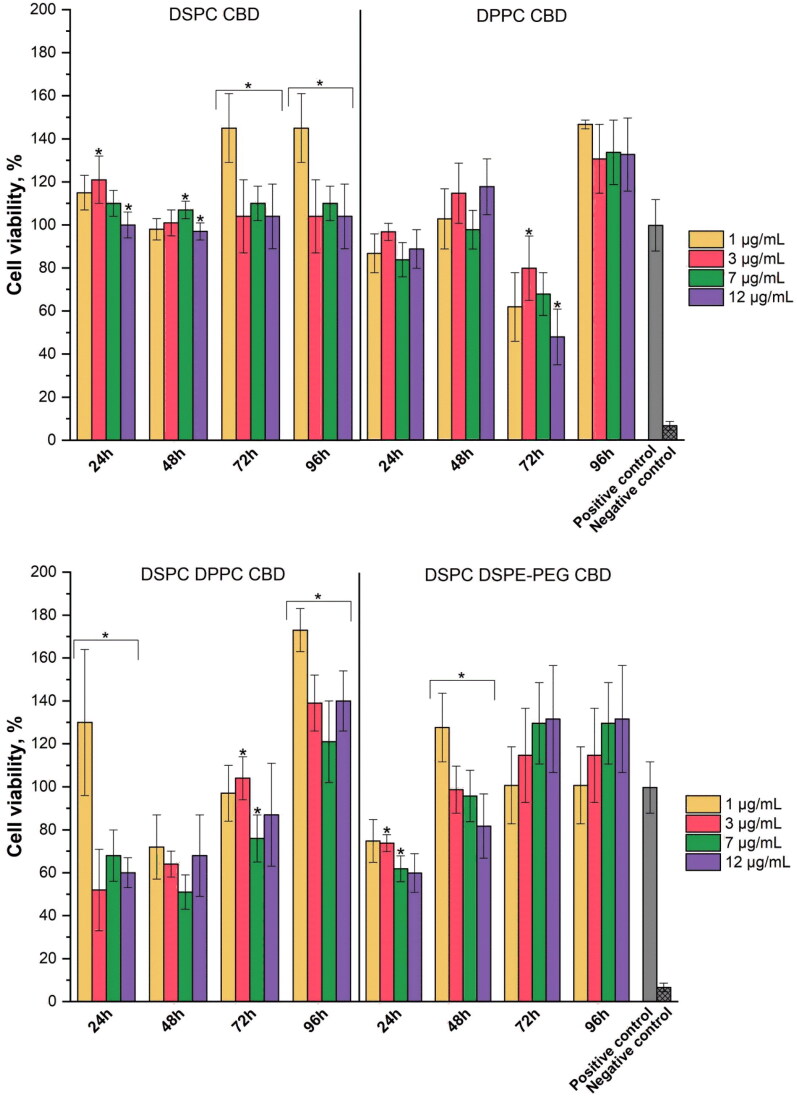
The influence of CBD liposomes on GMSCs viability after 24, 48, 72, and 96 h (*p < .05 between liposome samples, *n* = 6).

The cell viability results of CBD liposomes in Figure 6 vary between the concentrations and compositions of the liposomes, as well as the time. For all DSPC CBD liposome samples, the viability of GMSCs is over 70%, which according to the ISO 10993-5:2009 indicated that in the tested concentrations they are not cytotoxic. Other formulations of liposomes show cytotoxic effect at different time points – DPPC CBD after 72 h, DSPC DPPC CBD after 24 and 48 h, and DSPC DSPE-PEG CBD after 24 h. There is no clear trend between the liposome concentrations that is directly linked to CBD concentration (see Table 2), but at the highest liposome concentrations (7 and 12 mg/mL) reduced cell viability was observed more often than at lower liposome concentrations. Overall, the highest GMSCs viability was achieved after 96 h for all CBD liposome formulations. All liposomes without CBD showed no reduction of cell viability (see Supplementary material).

**Table 2.** CBD concentrations in the cell viability study.

Liposome sample name	Liposome concentration			
	CBD concentration (µg/mL/µM) in liposome samples used for *in vitro* study			
	1 µg/mL	3 µg/mL	7 µg/mL	12 µg/mL
DSPC CBD	0.15/0.48	0.5/1.4	1.1/3.3	1.8/5.7
DPPC CBD	0.13/0.41	0.4/1.2	0.9/2.9	1.6/5.0
DSPC DPPC CBD	0.16/0.51	0.5/1.5	1.1/3.6	1.9/6.1
DSPC DSPE-PEG CBD	0.17/0.54	0.5/1.7	1.2/3.9	2.1/6.6

## Discussion

For the application of CBD in the treatment of oral diseases for inflammation and pain management, it is essential to assess its effect on healthy cells found in oral cavity, like GMSC’s. These cells are easily isolated from healthy or inflamed gingiva and expanded *in vitro*, and they also possess multipotent differentiation potential (Zhang et al., 2009). Numerous research has been conducted with different cell lines. Gu et al. observed that CBD is cytotoxic to human monocytes and human telomerase-immortalized gingival keratinocyte cells at a concentration of 10 µg/mL (Gu et al., 2019). Another cytotoxic effect was observed on keratinocytes with CBD concentration 25–100 µM in ethanol (Jastrząb et al., 2019). A reduced viability was found for human bronchial epithelial cell line (BEAS-2B) with 31.8 µM of CBD solution (Muthumalage & Rahman, 2019). No effect was observed for human mesenchymal stem cells (MSCs) with 3 µM CBD in 0.01% ethanol (Schmuhl et al., 2014) and for human gingival MSCs with 5 µM CBD in 0.1% DMSO (Chiricosta et al., 2019). In this study, reduced cell viability was observed at lower CBD concentrations (Table 2) than in other research mentioned above. For some DSPC DPPC CBD and DSPC DSPE-PEG CBD liposome samples, the effect was even cytotoxic (with liposome sample concentration 7 and 12 µg/mL). High concentrations of cannabinoids can cause mitochondrial dysfunction by inhibiting respiratory electron transport chain activity, resulting in reduced electron transfer from cytochrome c to oxygen, leading to increased levels of reactive oxygen species and decreased adenosine triphosphate production, which together can increase cell death (Malheiro et al., 2023; Podinic et al., 2024). The obtained results show higher CBD bioavailability because liposomes have the ability to transfer the active compounds directly into the cell via cellular internalization (Li et al., 2022; Gandek et al., 2023). Similar effects have been seen with other hydrophobic compounds when incorporated into liposomes. Incorporating curcumin and doxorubicin into liposomes enhanced their effectiveness, with curcumin showing improved cellular uptake for pulmonary delivery, and doxorubicin achieving twice the oral bioavailability (Daeihamed et al., 2017; Bender et al., 2024). Therefore, if using liposomes as a drug delivery system, lower CBD concentrations could be needed to achieve the desired effect. The cell viability can exceed 100%, particularly at lower liposome concentrations (1 and 3 mg/mL), suggesting a promotion of cell proliferation by liposomes and/or CBD.

The achieved encapsulation efficiency in this study is slightly lower compared to available literature. Lecithin-based liposomes achieved 90% CBD encapsulation efficiency in studies by Valh et al. (2020) and Sedlmayr et al. (2023) soy-phosphatidylcholine liposomes with and without Tween 80 demonstrated an average encapsulation efficiencies of 92 and 94%, respectively (Franzè et al., 2022). The highest reported efficiency was achieved using archaesomes, a type of liposome prepared from ether lipids, with 97% encapsulation (Sedlmayr et al., 2023). However, an important distinction in our research is the higher CBD amounts used during liposome preparation process, achieving a theoretical CBD concentration of 1.0 g/L. In comparison, for soy-phosphatidylcholine liposomes, the maximal theoretical concentration was 0.5 g/L (Franzè et al., 2022), and for lecithin-based liposomes and archaesomes, the concentrations were 0.1 g/L and 0.5 g/L, respectively (Sedlmayr et al., 2023). This indicates that while encapsulation efficiency in our formulations is slightly lower, the ability to encapsulate higher CBD concentrations highlights the potential of our prepared liposomes.

The release of CBD has been investigated in various conditions, depending on the intended application of the liposomes. In the research of Zapata et al. (2023), lecithin-based CBD nanoliposomes were investigated in buccal (0–5 h), stomach (5–130 h), and duodenal (130–300 h) conditions, achieving 19%, 43%, and 100% of CBD release, respectively. Whereas Fu et al. (2022) investigated CBD release kinetics in PBS solution, using liposomes prepared from phosphatidylcholine and 20(S)-protopanaxadiol with CBD concentration 15 mg/kg. The results showed ∼20% release within the first hour and 50% of CBD release within the first 12 hours, followed by a sustained release up to 90% after 144 h (six days). In our research, the release was observed for a much longer period (504 h) but achieved 79–83% release of encapsulated CBD. Liposomes DSPC DSPE-PEG CBD showed the highest CBD encapsulation efficiency but CBD release in % was lower than for other samples. The presence of PEGylated lipids improves the stability of liposomes (Izumi et al., 2024) that can be seen also in DLS data; therefore, the release of CBD is slower.

Despite having lower CBD release at 24 h, DSPC DSPE-PEG CBD showed reduced cell viability, similar to DSPC DPPC CBD (except at liposome concentration 1 µg/mL) that had slightly higher CBD release. Because of having much smaller particles than DSPC DPPC CBD and other liposomes, DSPC DSPE-PEG CBD can have higher cellular internalization, leading to higher delivery of the active compound (CBD) directly into the cell (Augustine et al., 2020), which could cause reduced cell viability. Similar results can be observed with DSPC DPPC CBD liposomes. Despite having more sustained CBD release than DPPC CBD formulation, DSPC DPPC CBD liposomes show cytotoxic effect at 24 and 48 h time points, which could be explained due to slightly smaller particles than DPPC CBD, since size is one of the most important factors of liposome cellular uptake (Choi et al., 2023). Although DPPC CBD liposomes show the highest CBD release concentrations, the cytotoxic CBD effect was observed only after 72 h. For DSPC CBD liposomes, the cytotoxic effect was not observed, indicating consistent and sustained CBD delivery to the cells, which could be explained with the largest particle size. After 96 h, the average cell viability is above 100% for all CBD liposome formulations, indicating that the released CBD concentrations have no cytotoxic effect on the GMSCs.

Current pain management therapies, especially for chronic inflammatory pain, often rely on opioids, which carry the risk of addiction and many side effects (Dydyk & Conermann, 2024). Cannabinoids such as CBD provide a non-addictive alternative with fewer side effects, but effective clinical translation is hampered by poor solubility and rapid hepatic metabolism. Encapsulating CBD in liposomes improves the bioavailability of CBD by protecting it from immediate breakdown. For example, PEGylated DSPC liposomes had a sustained release profile (reaching 79% release in 504 hours) that could provide long-lasting pain relief without frequent dosing. For neurological conditions such as multiple sclerosis (Furgiuele et al., 2021), CBD has shown promise in reducing seizures and neuroinflammation, offering a new therapeutic avenue for these debilitating disorders. CBD liposomal formulations may further enhance its therapeutic efficacy in neurological conditions by providing better penetration of the blood–brain barrier, sustained drug release, and targeted action in affected tissues. Our results show that DSPC DSPE PEG liposomes significantly improve the encapsulation efficiency of CBD (87%), and this sample exhibits minimal cytotoxicity (Figure 6), indicating that it could be safely used in the nervous system.

Although our study highlights the advantages of DSPC, DPPC, and DSPC-DSPE-PEG liposomes in terms of CBD encapsulation and release profiles, further *in vivo* studies are needed to confirm these advantages in clinical settings. Future studies should investigate the biodistribution in specific tissues and the effects of CBD on target cells in inflammatory and neural environments. Furthermore, variability in cell viability, especially with different CBD concentrations, highlights the need to optimize doses for specific clinical applications to balance efficacy and safety.

## Conclusions

CBD was successfully incorporated into different compositions of liposomes. The encapsulation of CBD into the liposomes led to more compact and potentially more stable particles, since liposome size is one of the main factors that influence their stability. The presence of PEGylated lipid in the liposome resulted in the smallest liposome particles and the highest encapsulation efficiency. DSPC DSPE-PEG CBD liposomes also provided excellent stability and sustained release profile suitable for long-term therapeutic use.

The cell viability results indicated that by using liposomes as CBD delivery system, higher CBD bioavailability can be achieved due to liposome-mediated cellular internalization, suggesting that lower CBD concentrations might be needed if encapsulated in the liposomes to ensure the desired effect. The results also indicated that for liposomes with smaller particles higher cellular uptake occurred.

## Supplementary Material

Supplemental material.docx

## Data Availability

The data that support the findings of this study are available from the corresponding author [A. Dubnika], upon reasonable request.
