# Supplementary material for: Exploring the effects of cannabidiol encapsulation in liposomes on their physicochemical properties and biocompatibility
Source: Drug Deliv. 2025 Feb 6;32(1):2460666. doi: 10.1080/10717544.2025.2460666 (PMC11809167; doi:10.1080/10717544.2025.2460666)
Supplement: Supplemental material.docx [file IDRD_A_2460666_SM4715.docx]

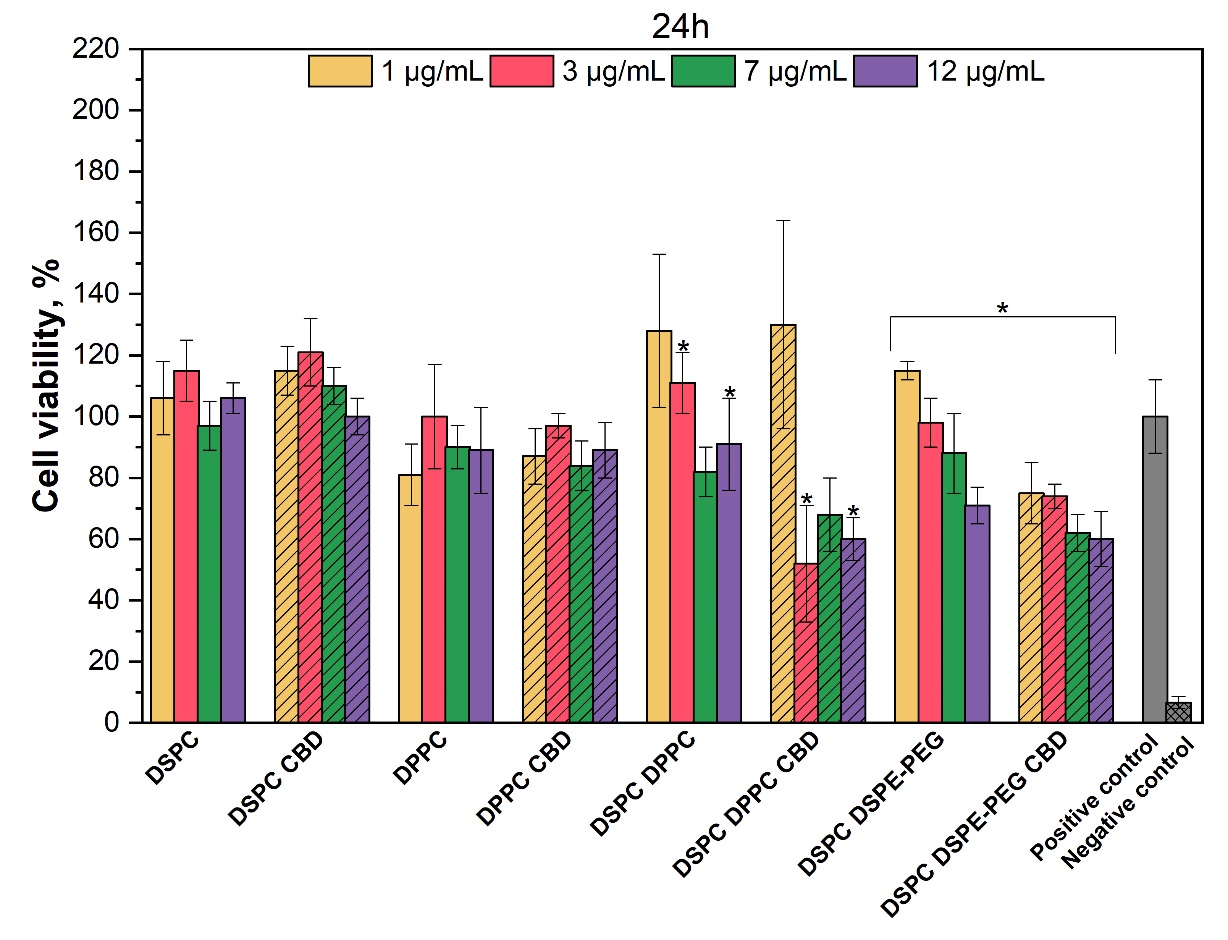


**Fig. S1.** The influence of liposomes with and without CBD on GMSCs viability after 24 h


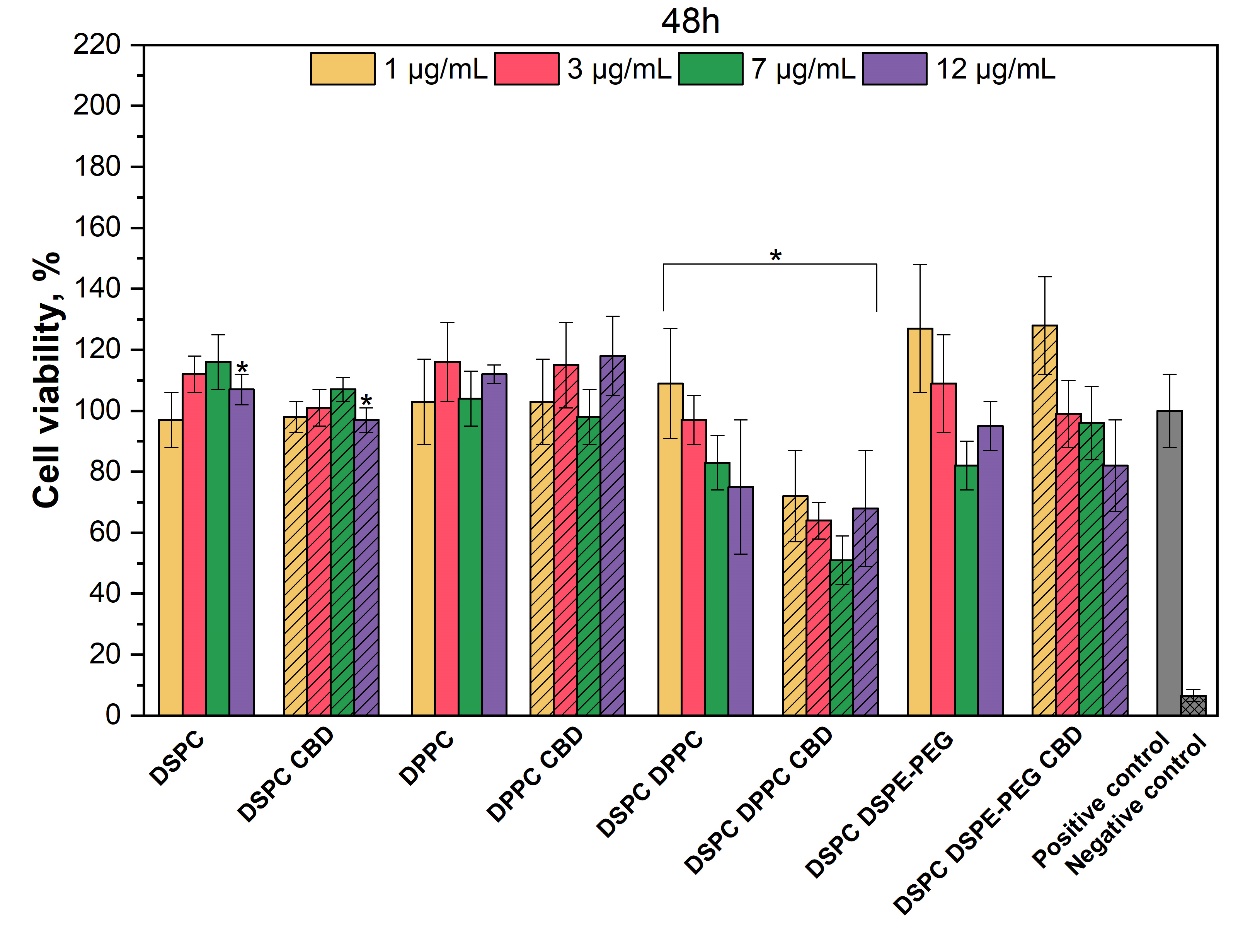


**Fig. S2.** The influence of liposomes with and without CBD on GMSCs viability after 48 h


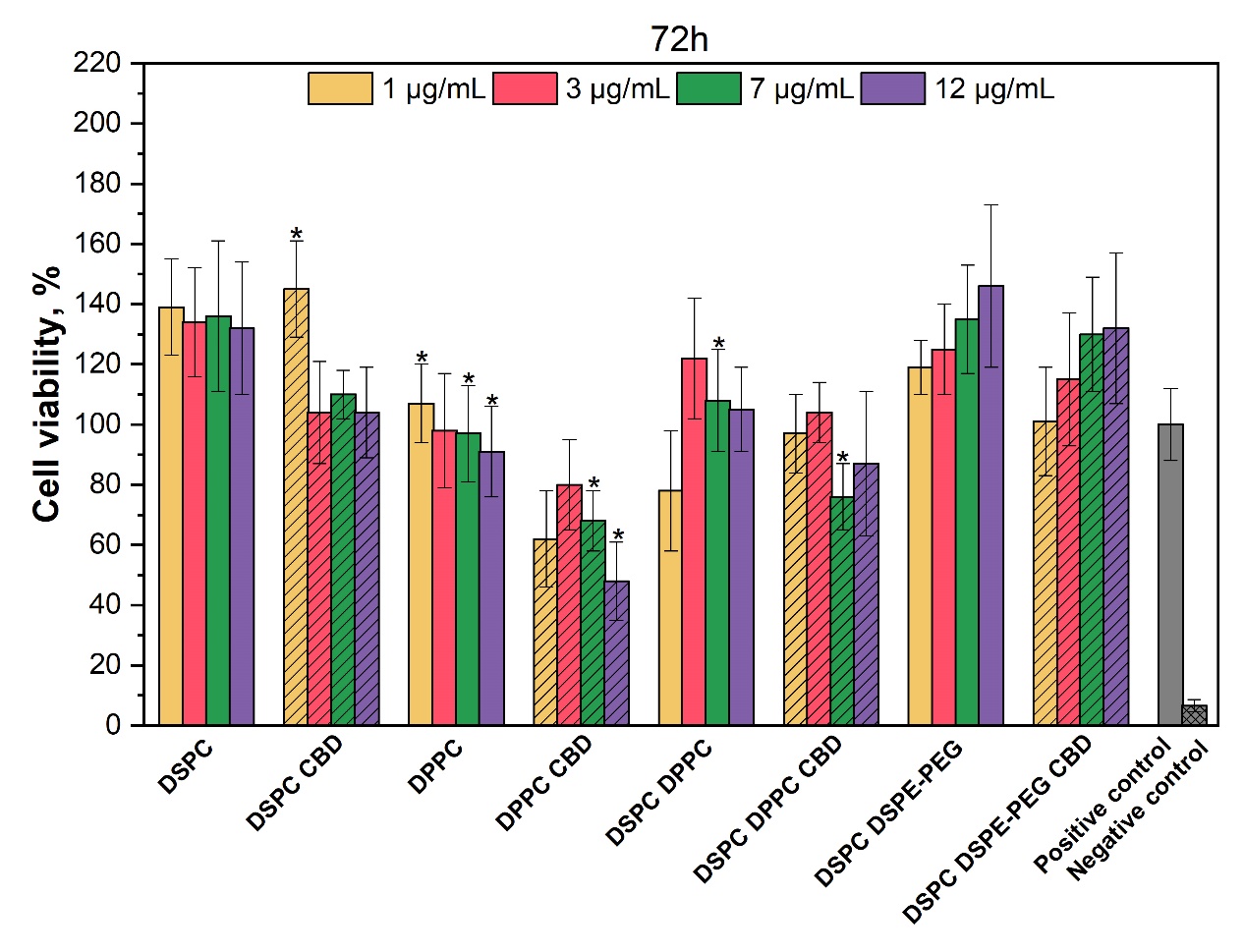


**Fig. S3.** The influence of liposomes with and without CBD on GMSCs viability after 72 h


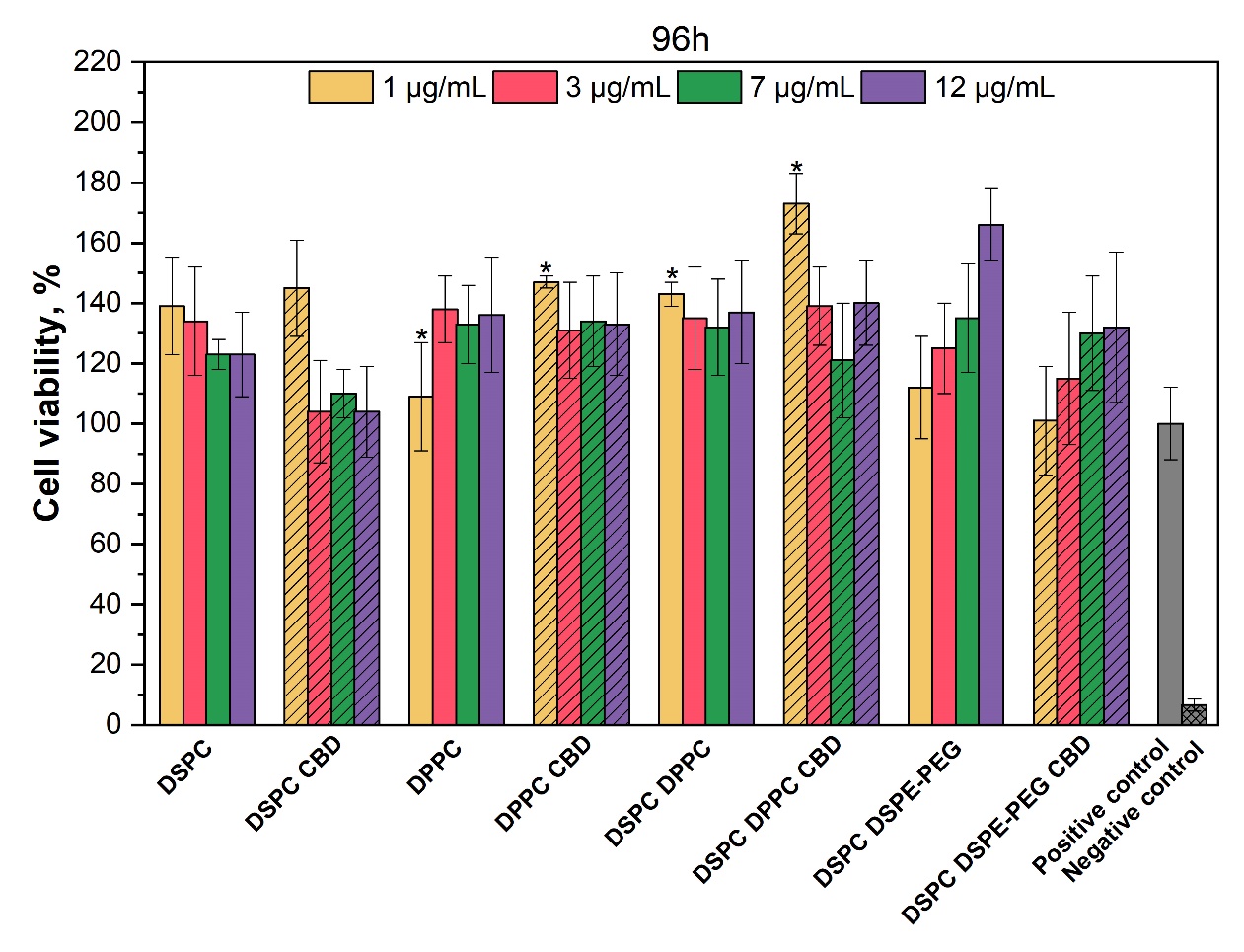


**Fig. S4.** The influence of liposomes with and without CBD on GMSCs viability after 96 h
